# Increased anticipatory brain response to pleasant touch in women remitted from bulimia nervosa

**DOI:** 10.1038/s41398-020-00916-0

**Published:** 2020-07-16

**Authors:** Christina E. Wierenga, Amanda Bischoff-Grethe, Laura A. Berner, Alan N. Simmons, Ursula Bailer, Martin P. Paulus, Walter H. Kaye

**Affiliations:** 1 Department of Psychiatry, University of California, San Diego, CA USA; 2 VA San Diego Healthcare System, San Diego, CA USA; 3 Division of General Psychiatry, Department of Psychiatry and Psychotherapy, Medical University of Vienna, Vienna, Austria; 4 Laureate Institute for Brain Research, Tulsa, OK USA

**Keywords:** Neuroscience, Human behaviour, Psychiatric disorders

## Abstract

Bulimia nervosa (BN) is characterized by affective instability and dysregulated behaviors (binge eating, fasting, self-induced vomiting) that disrupt bodily homeostasis. Mechanisms underlying dysregulation in BN are unclear, although altered reward responsivity, anticipatory processing of environmental cues, and interoception (detection and integration of body-state signals to regulate behavior) have been implicated in BN pathophysiology. We aimed to determine whether BN is associated with ineffectively predicting body state or integrating predicted experience with actual experience by examining neural response to anticipation and experience of affective touch, a pleasant interoceptive stimulus that acts on sensory and emotional systems to guide behavior. During fMRI, we administered soft strokes to the palm and forearm in women remitted from BN (RBN; *N* = 23) and control women (CW; *N* = 25). A Group (RBN/CW) × Condition (anticipation/touch) interaction was found in the right dorsal caudate; both CW and RBN had increased activation during touch compared with anticipation, with RBN demonstrating marginally greater anticipatory response than CW. For RBN, those individuals who showed greater anticipatory response in the dorsal caudate also reported higher levels of harm avoidance. RBN individuals relative to CW showed greater activation in left putamen and insula during the anticipation but not when experiencing an affective touch. This increase during anticipation rather than the actual experience of the affective touch is consistent with a top-down preparatory process which is associated with harm avoidance and is similar to what has been observed in anxious individuals. This aberrant signal integration could disrupt feedback processing, serving to maintain disordered behavior.

## Introduction

Bulimia nervosa (BN) is characterized by emotion dysregulation and recurrent episodes of binge eating and compensatory behaviors, like fasting and self-induced vomiting, motivated by a drive for thinness, fear of fatness, and body image dissatisfaction. These behaviors are thought to reflect increased appetitive motivation, altered anticipatory processing of environmental cues and a disruption of bodily homeostasis, implicating reward processing, anticipation, and interoception in the pathophysiology of BN^1,2^. Interoception refers to the sensing and integration of body-state signals that gives rise to emotions, determines one’s experience of the body and organizes behavior to meet one’s physiological needs^2–5^. A growing literature suggests an important role of the experience of the body in the emergence and maintenance of eating disorders^2,6–8^, with impairments in interoception representing a transdiagnostic feature of eating disorders hypothesized to be related to disrupted interoceptive neural processing^9^. Interoception was traditionally considered to relate to internal signals originating from the body (e.g., visceral signals, heartbeat, breathing rate) but more recent conceptualizations include broader central nervous system physiological representations such as body temperature, pain, itch, and affective touch^9–11^.

BN has been associated with decreased self-reported interoceptive awareness^12,13^, reduced sensitivity to pain^14–17^ and gastric distention^18,19^, and poor interoceptive accuracy^7^ (although heartbeat detection findings are mixed^20^). However, most studies have neglected to examine anticipatory aspects of interoception, despite the important role of context needed to properly evaluate the salience and value of interoceptive experience, which can only be achieved through anticipatory and predictive framing. Given evidence of altered anticipatory response to food cues in BN^21^, it is possible that BN may reflect the experience of ineffectively predicting body state or integrating predicted experience with actual experience. While there is growing evidence for altered fronto-striatal circuits associated with altered reward anticipation and processing in BN^21–23^, research has focused solely on monetary reward (non-interoceptive, non-eating disorder-specific) and pleasant tastes (interoceptive but eating disorder-specific) with limited attention to interoceptive and non-eating disorder-specific reward processing. Thus, little is known about underlying functional neurocircuit alterations that might give rise to non-eating-related interoceptive disturbances in BN.

A mismatch between anticipated and experienced outcomes (aka, prediction error) is thought to contribute to maladaptive behavior such as impulsivity or pathological avoidance, and could contribute to dysregulated motivated behavior in BN, such as excessive over- and under-consumption of food^24,25^. Notably, the discrepancy between decreased interoceptive accuracy (heartbeat detection) and elevated interoceptive sensibility (subjective sensitivity to internal sensations), interpreted as a trait interoceptive prediction error, has been shown to predict anxiety in autism^26^. In eating disorders, altered brain response to the expectancy and receipt of palatable food^21^ or food cues^23^ has been reported in individuals with bulimic episodes. The insula is a hub for the evaluation of interoceptive signals, including taste, pain, touch, and visceral sensations, playing a critical role in both the anticipation and processing of body-state information that guides behavior^10^. Processing the receipt or experience of interoceptive input occurs in the mid-to-posterior insula^27^, whereas anticipation of interoceptive stimuli is localized to the anterior insula. This is consistent with the notion that the anterior insula has a “preparatory function”^27^ and integrates signals related to the physiological condition of the body with affective and motivational drives^28^. The insula projects to the ventral striatum, which is involved in identifying rewarding and emotionally significant stimuli^29,30^. The ventral striatum guides motivated behaviors and decision-making via its connections with the dorsal cognitive neural circuit, which includes the dorsal caudate, dorsal anterior cingulate, and ventrolateral and dorsolateral prefrontal cortex^31^. In particular, the dorsal caudate is associated with cognitive control and reward anticipation and evaluation, including the subjective experience of craving, wanting and desire, particularly for natural (e.g., food) versus artificial (e.g., drugs) rewards^32–37^. Dysfunction within this fronto-striatal network is thought to be a key neural mechanism underlying altered behavioral and reward regulation in eating disorders^38,39^.

Neuroimaging research examining altered interoception in BN is scant. Existing studies have focused primarily on symptom-specific (e.g., taste, hunger) or unpleasant (e.g., restrictive breathing) interoceptive signaling. For example, increased reward response in BN to pleasant tastes^22,38,40–47^ and decreased response to aversive taste^22^ has been reported, with one study showing a lack of modulation of taste reward response by satiety^43^. To our knowledge, we conducted the only non-food-specific interoceptive imaging study in BN, which examined interoceptive response before, during, and after restricted breathing events^48^. We found that women remitted from BN (RBN) had increased activation during anticipation of breathing load with an aberrant decline in neural activation over the course of the aversive experience in a network associated with processing interoceptive state changes, including the mid-insula and cortico-striatal regions. These findings suggest that altered incentive processing, characterized by excessive anticipatory activation before and an abnormal decline in activation during non-painful aversive interoceptive events, may interfere with the ability to integrate expectations about homeostatic state changes with the actual experiences of these changes, contributing to BN symptoms that disrupt homeostasis, such as binge eating and purging^48^.

Despite known alterations in food and money-specific anticipation, processing, and learning^22,23,39–41,47,49^, very little is known in relation to non-symptom-specific, pleasant interoceptive events in BN. To determine whether BN is associated with a generalized (non-food-related) deficit in interoception, we examined whether RBN and control women (CW) differ in brain response to anticipation or experience of pleasant affective touch. Women remitted from BN were studied to avoid confounding physiological effects of active symptoms (e.g., malnutrition, electrolyte imbalances) with brain alterations and maintain consistency with other studies in remitted samples^43,48,50,51^. Affective touch is a pleasant interoceptive stimulus^10,11,52,53^. Affective touch acts on both sensory and emotional systems via different afferent fibers in the skin [e.g., palmar A∝-fibers that relay sensory tactile information, C tactile (CT) fibers that convey sensory and hedonic information] to promote an awareness of one’s body, and also help guide behavior and social interactions^8,54–57^, though a recent study suggests these systems may not be as divergent as previously thought^58^.

We previously showed women remitted from anorexia nervosa (RAN) had reduced brain response in the mid-insula during both anticipation of touch and aversive breathing restriction relative to CW, but a greater response when experiencing these stimuli, suggesting an impaired ability to predict and interpret incoming physiological stimuli in AN^50,51^. Although AN and BN share several overlapping symptoms and traits (e.g., anxiety, body image disturbance), they are also characterized by divergent traits (e.g., avoidance vs approach; inhibited vs impulsive) that might suggest opposite patterns of response to the anticipation and experience of interoceptive stimuli. Thus, we predicted individuals with RBN would also demonstrate a mismatch between response to the anticipation and the experience of touch, but in the opposite direction of the AN study and similar to our prior study of aversive breathing in BN^48^, with increased anticipatory touch response and decreased response to the experience of touch compared with CW in striatal and insula reward and interoception regions. In RAN, lower anticipatory touch response was associated with higher levels of harm avoidance, greater body dissatisfaction, and higher perceived touch intensity ratings^51^. Similarly, we expected altered anticipatory touch response would be associated with increased harm avoidance in RBN, given that harm avoidance reflects a transdiagnostic feature of eating disorders^59–61^.

## Materials and methods

### Participants

After excluding three women remitted from BN (RBN) and one control woman (CW) with incomplete or unusable imaging data, our final sample included 23 RBN based on *DSM-IV* criteria (11 with a prior history of anorexia nervosa) and 25 demographically-matched healthy CW. Data from CW were previously reported in a similar study of anorexia nervosa^51^. Remittance was defined as the absence of binge eating, purging, restrictive eating behaviors, and cognitive symptoms, occurrence of regular menstrual cycles, and a weight above 85% of ideal body weight with no fluctuations >3 kg for at least 1 year prior to the study^62^. RBN were recruited nationally, and CW were recruited locally. Women were excluded from the study if they met diagnostic criteria for a current *DSM-IV* Axis I diagnosis, took psychotropic medication within 3 months prior to study, had a history of alcohol or drug abuse or dependence 3 months prior to study, were left-handed, or reported any medical or neurologic concerns contraindicative to MRI. After providing subjects a complete description of the study, written informed consent was obtained. The University of California, San Diego Human Research Protections Program approved all procedures.

### Procedures

#### Clinical measures

Current and past psychiatric history were assessed using the MINI International Neuropsychiatric Interview by Master’s level assessors^63^. Participants also completed the Eating Disorders Inventory-2 (EDI-2)^64^, Spielberger State-Trait Anxiety Inventory (STAI)^65^, the Temperament and Character Inventory (TCI)^66^, and the Beck Depression Inventory-II (BDI-II)^67^. Participants completed pre- and post-fMRI visual analog scale (VAS) questionnaires that rated soft touch of the forearm and palm, respectively, from “0 – not at all” to “10 – extremely” on pleasantness, unpleasantness, and intensity.

#### Soft touch fMRI paradigm

Participants were scanned within the first 10 days (early follicular phase) of their menstrual cycles during a soft touch fMRI paradigm (Fig. S1). Gentle strokes with a soft boar bristle brush (OXO International Ltd., NY) were administered on 4 cm long regions of skin by a trained research assistant. Stimulation occurred on either the ventral surface of the left forearm, a region believed to contain dense mechano-receptive CT-fibers, or the palm, where these fibers are absent^68–70^. As in prior studies^51,71–73^, these regions were both pre-measured and pre-marked for consistency, and each soft brush stroke occurred at a velocity of 2 cm/s in a proximal to distal direction, standardized by an audio tone that was routed to the research assistant’s headphones. This velocity is within the optimal range (1–10 cm/s) for CT-fiber stimulation and has been previously shown to activate the posterior insula^68^. The force applied was equal to the brush’s weight (8 oz).

As in prior research^51^, during each of two task runs, participants completed a continuous performance task, whereby they were presented with a left- or rightward pointing arrow on a gray rectangular background (3 s). Subjects were asked to press the left or right button of a button box to indicate the direction of the arrow, using the index and middle fingers of the right hand. The arrow’s background changed color to indicate one of three conditions: (1) a gray background indicated the baseline condition, in which no stimulus was expected or administered, averaging 9 s (three consecutive arrow trials) in duration; (2) a yellow background (6 s) indicated anticipation of soft touch of the left forearm, such that the participant could expect with 100% likelihood a subsequent soft touch of the forearm; and (3) a blue background (6 s) indicated anticipation of soft touch of the left palm, such that the participant could expect with 100% likelihood a subsequent soft touch of the palm. Following the anticipatory periods, the soft touch condition would occur (3 s), whereby the brush was applied to the previously indicated location for the first 2 s of the trial. All participants were informed of the task structure and the meaning of the colored backgrounds prior to task performance. Across both runs, anticipation and soft touch occurred twenty times for each location (palm, forearm). Each run lasted 420 s. Response accuracy and reaction time on the continuous performance task were recorded from the onset of arrow presentation for all trials.

### Data analysis

#### Behavioral analysis

Group level statistical analyses were performed using R (http://www.r-project.org). Welch’s *t*-tests were used to compare groups on clinical variables and VAS ratings of soft touch. A linear mixed effects (LME) analysis determined whether there were any Group (RBN, CW) × Location (palm, forearm) interactions separately for pre-scan VAS ratings, with group as the between-subject variable and time as the within-subject variables. Performance on the continuous performance task was assessed with a LME model that examined reaction time differences, with group (CW, RBN) as the between-subject variable and Condition (anticipation, soft touch) and Location (palm, forearm) as the within-subjects variables. For all analyses, subject was treated as a random effect, with Group, Condition, and Location as fixed effects.

#### MRI statistical analyses

Imaging data (see Supplement for fMRI protocol detail) were analyzed with AFNI, FSL, and R statistical packages. Following preprocessing (see Supplement), statistical analyses were performed using a general linear model (GLM), with individual events modeled using AFNI’s SPMG3 function. Six motion parameters (3 rotations and 3 translations) were used as nuisance regressors to account for motion artifact. To examine whether RBN and CW differed in brain response to the anticipation and experience of affective touch, data were analyzed using a Group (CW, RBN) × Condition (anticipation, soft touch) × Location (palm, forearm) linear mixed effects approach with group (CW, RBN) as the between-subject variable and Condition (anticipation, soft touch) and Location (palm, forearm) as the within-subjects variables. For all analyses, subject was treated as a random effect, with Group, Condition, and Location as fixed effects. To further examine group differences in anticipation and experience of soft touch, we tested a simplified model that examined Group × Location separately for anticipation trials and for soft touch receipt trials. To limit multiple comparisons, we restricted our primary and secondary fMRI analyses a priori to two bilateral regions of interest (ROI; derived from the Harvard-Oxford atlas^74^) involved in the anticipation and experience of interoceptive and rewarding stimuli: (1) the insula in its entirety, and (2) a striatum ROI that included the caudate, putamen, and nucleus accumbens (Fig. S2). These ROIs were used as search regions for all fMRI analyses, with a peak voxel of *p* < 0.01 with a cluster threshold of *p* < 0.025 (Bonferroni corrected for two ROIs) required for significance. The spatial autocorrelation function (acf) option in AFNI’s 3dFWHMx estimated intrinsic smoothness. Minimum cluster sizes were calculated with AFNI’s 3dClustSim in order to guard against false positives. This approach employs non-Gaussian models and spatial autocorrelation functions and is more robust than traditional methods^75^. The required minimum cluster size was 270 µL (10 contiguous voxels) for each ROI. An exploratory whole brain analysis examined group differences in activation across the whole brain (peak voxel of *p* < 0.01, cluster threshold of *p* < 0.05, resulting in a minimum cluster size 837 µL [31 contiguous voxels]). For significant clusters, post hoc analyses were conducted using *lsmeans* from R’s multcomp package with Tukey’s all-pair comparisons, and the *p*-values were false discovery rate (FDR)^76^ adjusted. Sensitivity analyses were conducted to examine the potential confounding impact of past anorexia nervosa (AN), past Major Depressive Episode, and past substance use disorders by comparing RBN participants with and without these historical comorbidities on mean activation in clusters in our full-sample between-group LMEs.

#### Primary robust regression analyses

Based on previous associations between anticipatory touch response and harm avoidance in women remitted from AN^51^, voxelwise Huber robust regressions^77^ were conducted in R to examine the association of TCI harm avoidance with the mean percent signal change of the blood oxygen level dependent (BOLD) response. Within-group individual regressions were performed against the percent signal change for anticipation palm, anticipation forearm, soft touch palm, and soft touch forearm. As above, significant clusters were identified within each ROI search region using AFNI’s 3dClustSim for small-volume correction with a peak voxel of *p* < 0.01. Results were Bonferroni corrected for two ROIs, and 4 touch task conditions (*α* < 0.006). To assess whether ROI-based clusters identified in the task-related LME analysis overlapped with those identified in the robust regression analyses of harm avoidance, we computed the intersection of the task-based clusters with those from the robust regression. Since both maps include only significant clusters, the resultant overlap may also be considered statistically significant^78^.

#### Exploratory regression analyses

Exploratory Huber robust regression analyses examined the relationship between neural activation and subjective VAS ratings for pleasantness (measuring positive valence) and intensity (measuring arousal). Due to non-normal distributions, VAS intensity predictors were natural log transformed and z-scored prior to regression. Exploratory Poisson robust regression analyses examined the relationship between neural activation and worst past self-induced vomiting and binge eating frequency within the RBN group only. Additional self-report ratings (i.e., TCI reward dependence, TCI novelty seeking, STAI state and trait anxiety, BDI depression, EDI-2 Body Dissatisfaction) and clinical variables (i.e., BMI, BN illness duration, duration of remission) were examined with Huber robust regression. As above, significant clusters were identified within each ROI using AFNI’s 3dClustSim for small-volume correction (per-voxel *p* < 0.01). To control for family-wise error, within-group analyses were also Bonferroni corrected for the number of clinical assessments and conditions tested (*α* < 0.003). Finally, the overlap of significant clusters identified with exploratory robust regressions of clinical variables with the primary Group × Condition × Location task-related LME analysis were also explored. The EDI-2 Interoceptive Awareness subscale was not examined because most participants (both CW and RBN) scored 0 on this measure.

## Results

### Participant characteristics

Groups did not differ significantly in age (RBN mean = 27.2; CW mean = 25.6; *p* = 0.4) or BMI (RBN mean = 22.0; CW mean = 22.2, *p* = 0.8; Table S1). Past mood and substance use disorders were more common in the RBN group, and the RBN group reported higher, but non-clinically significant, levels of depressive symptoms, state and trait anxiety, body dissatisfaction, and harm avoidance (Table S1).

### VAS scales

Post-scan VAS ratings were missing for one CW. Groups did not differ significantly on ratings of pre- or post-scan pleasantness, unpleasantness, or intensity of palm or forearm soft touch (*p*s > 0.1; Table S2). Groups also did not differ significantly in the change of pleasantness, unpleasantness, and intensity ratings of palm and forearm soft touch from pre- to post-scan (*p*s > 0.07; Table S2). In separate LME analyses, no significant main effects of Group or Location, and no significant Group × Location interactions were detected for either pre-scan or post-scan touch pleasantness, unpleasantness, and intensity ratings.

### Behavioral analyses

Five participants’ (3 CW, 2 RBN) behavioral responses on the continuous performance task were lost due to equipment failure. Groups did not differ significantly on task accuracy (*p*s > 0.2). For reaction time, there was a significant main effect of Location (*F*(1,41) = 5.7, *p* = 0.02) which suggested a slower response time for forearm compared with palm anticipation and touch across all participants. This suggests that processing of hedonic interoceptive information may require more cognitive resources and reduce vigilance more than processing sensory information. No other main effects or interactions were significant (*p*s > 0.1).

### Region of interest analyses

#### Main effect of Condition

**Fig. 1:**
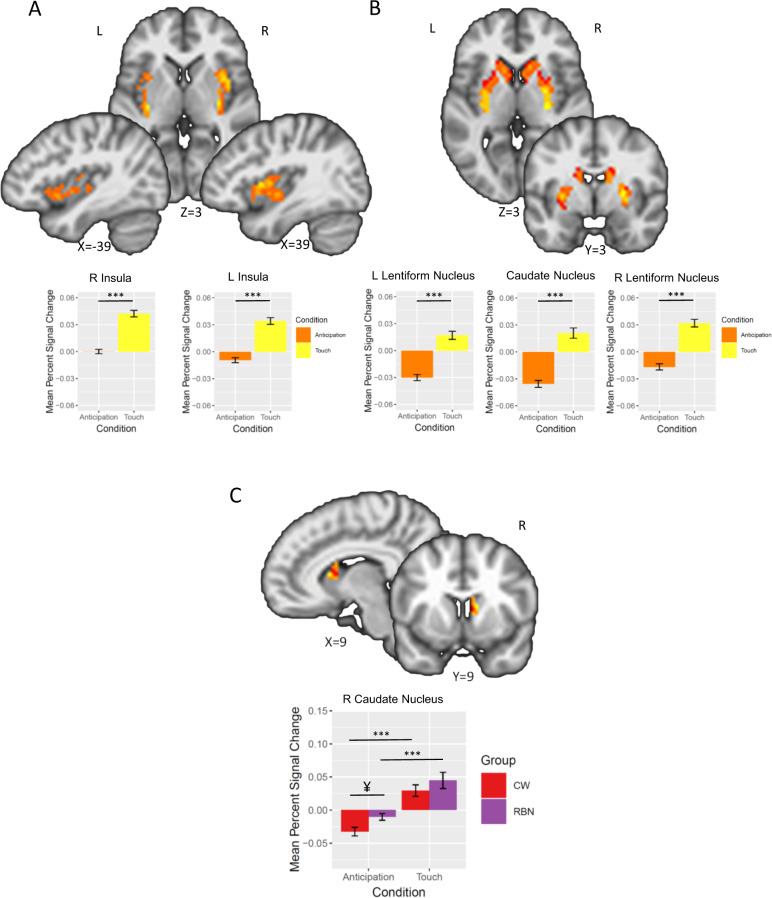
Results of the Group × Condition × Location linear mixed effects analysis within the bilateral striatum and insula demonstrating a main effect of Condition (anticipation, soft touch) and an interaction of Group × Condition for the soft touch paradigm. **a** Main effect of Condition (Anticipation, Soft Touch) within the bilateral insula. Overall, participants showed a greater BOLD response during soft touch relative to during anticipation. **b** Main effect of Condition within the bilateral dorsal striatum that included both the caudate and putamen. **c** Group (CW, RBN) × Condition (Anticipation, Soft Touch) interaction during performance of the soft touch paradigm within the right dorsal caudate. Both RBN and CW had a greater BOLD response during soft touch compared with anticipation, but RBN had a marginally greater BOLD response during anticipation compared with CW. Error bars represent standard error of the mean. CW healthy comparison women, RBN women remitted from bulimia nervosa, L left, R right. ^¥^*p* = 0.11; ***p < 0.001.

Results of the Group × Condition × Location LME revealed a main effect of Condition, such that across groups, both the bilateral insula, encompassing the anterior, middle, and posterior portions, and the dorsal striatum (centered in the putamen and including the dorsal caudate) showed a greater response during soft touch receipt than during anticipation (Table 1, Fig. 1a, b).

**Table 1:** Results of the Group × Condition × Location linear mixed effects analysis within the bilateral striatum and insula demonstrating a main effect of Condition (anticipation, soft touch) and an interaction of Group × Condition for the soft touch paradigm.

							Post hoc comparisons		
Region	L/R	Volume (voxels)	*X*	*Y*	*Z*	*F* value	Comparison	*z*	*p* (FDR)
MAIN EFFECT OF CONDITION									
Insula									
Insula	R	237	36	−2	4	34.85	Soft touch > Anticipation	6.29	<0.001
	L	231	−35	−8	4	34.44	Soft touch > Anticipation	5.85	<0.001
Striatum									
Putamen	L	400	−21	0	6	42.76	Soft touch > Anticipation	5.21	<0.001
	R	167	27	−4	2	55.34	Soft touch > Anticipation	5.76	<0.001
Caudate nucleus	R	214	14	7	14	26.91	Soft touch > Anticipation	4.63	<0.001
GROUP × CONDITION INTERACTION									
Striatum									
Dorsal caudate	R	18	10	11	10	7.73	CW: Soft touch > Anticipation	5.50	<0.001
							RBN: Soft touch > Anticipation	4.68	<0.001
							Anticipation: RBN > CW	1.77	0.11
							Soft touch: RBN > CW	1.22	0.23

Note: RBN had greater anticipatory BOLD response than CW in the left dorsal putamen and insula. The posterior insula showed greater activation to touch of the palm compared with forearm. Center of mass coordinates reported in MNI space. Small-volume correction was determined with Monte-Carlo simulations (via AFNI’s 3dClustSim) to guard against false positives. Post hoc analyses were conducted using glht from the multcomp package in R to calculate general linear hypotheses using Tukey’s all-pair comparisons, and *p*-values were FDR adjusted.

*CW* healthy comparison women, *L* left, *R* right, *RBN* women remitted from bulimia nervosa.

#### Group × Condition interaction

Results of the Group × Condition × Location LME also revealed a significant Group × Condition interaction in the right dorsal caudate revealed that both RBN and CW had a greater BOLD response during soft touch compared with anticipation, but RBN had a marginally greater BOLD response during anticipation compared with CW (Table 1, Fig. 1c). No interactions were detected within the insula.

There were no significant findings within the insula or striatum for any other interaction (i.e., Group × Condition × Location, Group × Location, Condition × Location), or for the main effect of either Group or Location.

#### Planned contrasts: anticipation trials

**Fig. 2:**
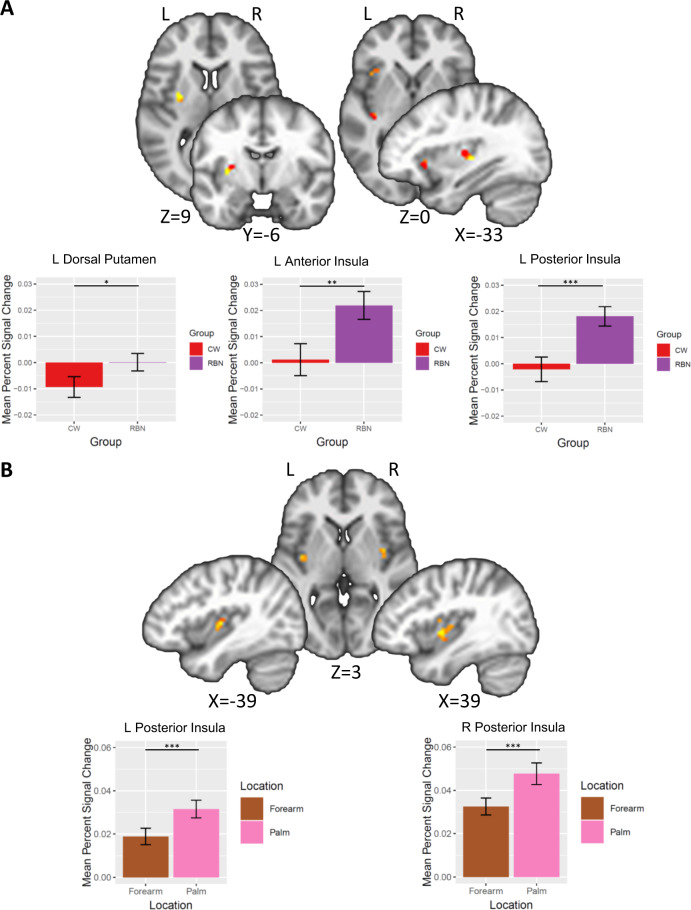
Results of Group (CW, RBN) × Location (hand, forearm) linear mixed effects analyses separately for anticipation trials and touch trials within the bilateral striatum and insula. **a** Anticipation trials. A Group×Location LME for anticipation trials revealed a main effect of Group in the left dorsal putamen and the left anterior and posterior insula, such that RBN had greater anticipatory BOLD response than CW. **b** Touch Trials. A Group×Location LME for touch trials revealed a main effect of Location in the bilateral posterior insula, with greater BOLD response to touch of the palm compared with forearm. Error bars represent standard error of the mean. *p < 0.05, **p  <0.01, ***p < 0.005. ***p < 0.005.

A Group × Location LME for anticipation trials revealed a main effect of Group in the left dorsal putamen and the left anterior and posterior insula, such that RBN had greater anticipatory BOLD response than CW. There were no significant findings within the insula or striatum for the main effect of Location or the interaction of Group × Location (Table 2, Fig. 2a).

**Table 2:** Results of Group (CW, RBN) × Location (hand, forearm) linear mixed effects analyses separately for touch anticipation trials and touch receipt trials within the bilateral striatum and insula.

Region	L/R	Volume (voxels)	*X*	*Y*	*Z*	*F* value	Comparison	*z*	*p*(FDR)
ANTICIPATION TRIALS									
*Main effect of Group*									
Striatum									
Dorsal putamen	L	13	−31	−6	6	10.3	RBN > CW	2.0	0.049
Insula									
Posterior insula	L	15	−35	−20	4	11.07	RBN > CW	2.9	0.003
Anterior insula	L	10	−36	21	−3	10.26	RBN > CW	2.6	0.009
TOUCH TRIALS									
*Main effect of Location*									
Insula									
Posterior insula	R	23	39	−5	1	20.17	Palm > Forearm	2.97	0.003
Posterior insula	L	14	−38	−12	6	16.38	Palm > Forearm	2.87	0.004

Note: RBN had greater anticipatory BOLD response than CW in the left dorsal putamen and insula. The posterior insula showed greater activation to touch of the palm compared with forearm. Center of mass coordinates reported in MNI space. Small-volume correction was determined with Monte-Carlo simulations (via AFNI’s 3dClustSim) to guard against false positives. Post hoc analyses were conducted using glht from the multcomp package in R to calculate general linear hypotheses using Tukey’s all-pair comparisons, and *p*-values were FDR adjusted.

*CW* healthy comparison women, *L* left, *R* right, *RBN* women remitted from bulimia nervosa.

#### Touch receipt trials

A Group × Location LME for touch trials revealed a main effect of Location in the bilateral posterior insula, with greater BOLD response to touch of the palm compared with forearm. There were no significant findings within the striatum for the main effect of Location, and no significant findings within the insula or striatum for the main effect of Group or the interaction of Group × Location (Table 2, Fig. 2b). Results of additional sensitivity analyses indicated that there were no significant differences in activation between participants with and without past AN, Major Depressive Episode or substance use disorder.

### Exploratory whole brain analyses

#### Main effect of Group

There was a significant main effect of Group, with RBN showing a greater BOLD response than CW collapsed across trial types, within regions of the frontal and parietal lobes, including the left inferior and medial frontal gyrus and the left precuneus and inferior parietal lobule (Table S3).

#### Main effect of Condition

There was a significant main effect of Condition, with multiple clusters throughout frontal, temporal, parietal, and occipital regions showing a greater response during soft touch receipt relative to anticipation (Table S3).

#### Main effect of Location

There was a main effect of Location within the right postcentral gyrus, with all participants showing a greater response to the palm compared with the forearm (Table S3).

#### Group × Condition interaction

A Group × Condition interaction was detected within the bilateral cuneus, left precuneus, and right superior frontal gyrus, such that CW showed a greater response during soft touch compared with anticipation. In the right superior frontal gyrus, RBN showed a greater response to anticipation compared with CW (Table S3).

There were no significant findings for any other interaction (i.e., Group × Condition × Location, Group × Location, Condition × Location).

### Primary robust regression analyses

**Fig. 3:**
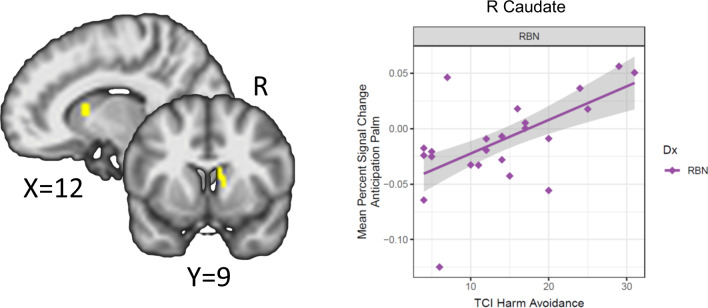
Association between brain activity in the dorsal caudate for the Group × Condition interaction with TCI harm avoidance for the RBN group. RBN with higher harm avoidance had higher BOLD response during anticipation of touch of the palm [*t* = 4.6, *r* = 0.60, *p* = 0.003] in the right dorsal caudate (peak coordinates: *X* = 10, *Y* = 11, *Z* = 10), as identified by Huber robust regression (total cluster size = 64 voxels; number of voxels in the robust regression significant cluster which overlap with voxels in the Group × Condition interaction cluster = 15 voxels). RBN women remitted from bulimia nervosa, TCI Temperament and Character Inventory.

RBN with higher harm avoidance scores had higher BOLD responses during palm touch anticipation in the right dorsal caudate (Fig. 3). Moreover, this harm avoidance-associated cluster overlapped with the Group × Condition cluster, suggesting that harm avoidance may have played a role in the increased anticipatory response in RBN. No associations between harm avoidance and BOLD response were detected in CW.

### Exploratory robust regression analyses

#### VAS ratings

Analyses suggested that CW with higher pleasantness ratings had higher BOLD responses in the left nucleus accumbens during anticipation of touch of the palm (Table S4). No associations between BOLD response and VAS ratings were detected in RBN.

#### Clinical measures

RBN with greater TCI reward dependence had higher BOLD response in the left anterior insula during forearm touch, and RBN with longer illness durations had decreased BOLD response during palm touch in the left putamen (Table S4). No other associations between BOLD response and clinical measures were detected in RBN.

## Discussion

This is the first neuroimaging study to examine neural response to interoceptive expectancy and experience of affective touch to determine whether BN is associated with ineffectively predicting body state or integrating predicted experience with actual experience. Results lend further support for the role of altered interoceptive expectancy in the pathophysiology of BN, and extend our prior findings to suggest these interoceptive alterations generalize across both aversive (restricted breathing) and pleasant (soft touch) stimuli. Specifically, the RBN group demonstrated an altered response to the anticipation of affective touch, a pleasant interoceptive stimulus that acts on both sensory and emotional systems and is thought to guide behavior and social interactions based on body awareness. Despite similar ratings of touch pleasantness between groups, we found an interaction of Group (CW, RBN) × Condition (anticipation, soft touch) in the right dorsal caudate; both groups showed greater response to receipt versus anticipation of a soft touch to either the forearm or palm, with a trend toward greater anticipatory response in RBN compared with CW. Planned follow-up contrasts revealed greater anticipatory touch response for RBN compared with CW in the left dorsal putamen and the anterior and posterior insula. No group differences in response to receipt of touch were detected in follow-up analyses. Of note, greater anticipatory palm touch response in the right dorsal caudate was associated with increased harm avoidance in RBN. Elevated interoceptive anticipatory response in the dorsal striatum and insula in RBN relative to controls, in the context of similar neural response to experienced touch, suggests difficulty integrating environmental cues with actual body–brain signals. This aberrant signaling might indicate altered temporal linkage between interoceptive and non-interoceptive (contextual) information that could disrupt the anticipated experience of one’s body, and impair adaptive learning from pleasant body-related signals.

Our findings of altered anticipatory touch signaling in RBN are consistent with Bayesian predictive coding accounts that posit that eating disorders result from a deficit in predictive multisensory body integration, i.e., the ability to integrate multimodal sensory data, internal body information, and predictions from prior body-related experiences^79,80^. According to this model, multisensory bodily representations and signals inform predictions about the causes of sensory events and drive one’s behavioral responses to these internal and external sensory events. The purpose is to unify these differing bodily signals by reducing prediction errors (or “surprise”) about the expected sensory input, and thus improve accuracy of predictions and plans for coping with them emotionally and behaviorally^81^. The interoceptive deficits observed in individuals with EDs have been hypothesized to reflect a deficit in this multisensory bodily integration that affects the ability to identify and correctly link interoceptive body signals to their potential pleasant or aversive consequences, and to modify stored information of body-related events accordingly^79,82–84^. This failure to functionally adapt to body-related experiences is thought to contribute to a distorted representation of one’s body, one of the predominant symptoms of eating disorders. Our findings reflect a potential mismatch between the neural response to anticipation and experience of affective touch, suggesting a deficit in the ability to correctly predict incoming physiological sensations and adjust expectations appropriately to drive adaptive coping of these bodily sensations in BN. Notably, we previously observed the opposite pattern of response (anticipation > experience) in women remitted from AN, suggesting that the directionality of the mismatch might underlie avoidance vs approach behaviors.

The localization of the Group × Condition interaction to dorsal striatal neural circuitry regulating cognitive control, and reward expectancy (e.g., craving) and evaluation^85,86^, adds to a growing literature implicating altered fronto-striatal networks, and the caudate nucleus in particular, in dysregulated inhibitory control^87–89^ and reward processing^89^ in BN. For example, regionally selective volume reductions of the caudate nucleus have been specifically implicated in BN^90–92^. Reduced activation of the caudate nucleus during skin stroking versus skin indentation has also been reported in AN^93^, suggesting altered function of the dorsal striatum influencing evaluation of pleasant tactile stimuli may be a transdiagnostic feature of eating disorders. Moreover, RBN individuals relative to CW showed greater activation in left putamen and insula during the anticipation but not when experiencing an affective touch. It is important to note that in our study, the anticipatory phase always predicted a pleasant touch, and thus we measured neural anticipation under conditions of certainty. This selective finding of altered anticipatory response is consistent with the notion that dysfunction in anticipatory processing is a fundamental component of the psychopathology of disorders characterized by excessive approach (e.g., addictive disorders) or avoidance (e.g., anxiety disorders) motivation^94^. This increased anticipatory signaling in RBN for predictable events likely reflects inefficient or ineffective preparation even for certain upcoming experiences.

We have recently proposed that interoceptive psychopathology arises from altered active inference due to disorder-specific expectations, which are the result of hierarchically based feedback and feedforward loops that are reinforced by mental rehearsal^95^. These expectations alter interoceptive perception that—in turn—changes how the internal and external environment affects the selection of adaptive behaviors. Moreover, we hypothesized that individuals with depression and anxiety exhibit two interoceptive dysfunctions. First, these individuals have overly strong expectations, i.e., hyperprecise priors, which shape the perception of the interoceptive afferences. Second, these individuals have difficulty adjusting these expectations when the internal or external environment changes, i.e., show context rigidity. In this disorder population individuals with RBN may have increased interpersonal anxiety, which results in exaggerated activation during the anticipation of affective touch.

Our finding of an association of greater anticipatory caudate response with elevated harm avoidance (e.g., anxiety) in RBN is consistent with this notion and adds to a growing body of evidence linking negative affect with increased caudate anticipatory reward response in BN, perhaps providing a mechanism for behavioral findings that bulimic episodes are often preceded by and planned in the context of negative affect and emotional instability^96–98^. In fact, several studies have found direct associations between caudate function/structure and clinical symptoms in BN, including negative affect^21^, craving^99^, and self-induced vomiting frequency^92^. The relationship between reward function in the caudate and harm avoidance in eating disorders is further supported by positron emission tomography (PET) studies showing that dopamine binding in the dorsal caudate is related to increased anxiety and harm avoidance in RAN and RBN^100,101^.

The exploratory finding that greater reward dependence in RBN was associated with greater insula response to the receipt of forearm touch is consistent with prior findings that personality traits are linked to salience network (e.g., insula and dorsal anterior cingulate cortex) activation^102^. Reward dependence is associated with behavior that is driven by positive motivation, such as social approval. Given the link between the salience network, reward and motivation, and interoception, it will be important for future research to explore whether the association between interoceptive response in the insula and reward dependence is related to impulsivity and addictive behavior that may maintain repeated maladaptive over-consumptive behaviors. Despite hypothesized links between interoception and body image^8^, we did not detect associations between brain response and EDI Body Dissatisfaction, perhaps due to a restricted range as a function of our inclusion/exclusion criteria.

### Limitations

This represents the first neuroimaging study of affective touch in BN. Our sample size was relatively small, and future studies with larger samples are needed to replicate results. Findings support altered interoceptive incentive processing in BN. However, because anticipatory cues in the affective touch task were 100% predictive of outcome, results reflect brain response to the anticipation and receipt only of predictable stimuli. We observed similar pleasantness ratings for stroking palm and forearm, which do not seem consistent with the distinction between A∝ sensory tactile and CT hedonic fibers. It is commonly observed that glabrous skin touch is also perceived as pleasant. We, and others, have failed to detect differences in perceived pleasantness between soft touch to the palm and forearm^51,71,103^, though distinctions between emotional and sensory discriminatory descriptors for the forearm and the palm have been reported^104^. The absence of a Group × Condition interaction in the insula was unexpected, given our prior findings of decreased insula response to touch anticipation and increased response to experience of touch in RAN^51^, and increased anticipatory insula response to restricted breathing in RBN^48^. However, data from healthy controls indicate that the insula is both functionally^105^ and structurally^106^ connected to the caudate nucleus, supporting the shared functions of the two regions. Although results provide support for a mismatch between neural responses for what is expected and experienced, the impact of this mismatch on learning and decision-making remains unknown. Similarly, further studies are needed to determine the meaningfulness of potential lateralized findings for touch anticipation in BN. We also found greater activation in the posterior insula for stroking on the palm vs the forearm, which conflicts with a meta-analysis that revealed the posterior insula was more likely to be activated for affective touch^107^. However, a recent study in healthy volunteers suggests the insula processes both affective touch as well as discriminative touch^93^, supporting the elevated palm response reported herein. Studies in alcohol^72^ and substance^71,108^ users have failed to show a palm-forearm distinction, suggesting the insula may play a role in both kinds of touch. Finally, studying a remitted sample has the advantage of not confounding findings with physiological symptoms of the disease state (e.g., malnutrition, electrolyte disruption), but limits our ability to detect associations between brain response and active clinical symptoms. In addition, this approach does not resolve the concern of whether group differences reflect trait-based alterations that existed prior to disease onset or sequelae/brain changes resulting from maladaptive behaviors (e.g., caloric restriction, binge eating, purging). Replication in an ill sample is needed.

### Clinical implications

Altered interoception may have broad clinical relevance to BN as body–brain signaling guides decision-making, self-regulation, body image, and emotional experience, factors commonly disturbed in BN^2,8^. Altered interoceptive expectancy may contribute to dysregulated behavior related to affective physical sensations, and may lead to difficulty predicting and adapting to body-related experience. Elucidating the neurocircuitry contributing to BN symptoms may directly inform new therapeutics, and results lend support to the development and implementation of interventions that address altered interoceptive expectancy in BN (e.g., via modifying cue reactivity, reducing anticipatory anxiety through interoceptive exposure, and/or mindfulness). Alterations in internal signals for upcoming pleasant experiences may also indicate treatment strategies that increase reliance on external signals or feedback.

## Supplementary information

Supplementary Information

## References

[1] Klabunde M, Collado D, Bohon C (2017). An interoceptive model of bulimia nervosa: a neurobiological systematic review. J. Psychiatr. Res..

[2] Khalsa S, Lapidus R (2016). Can interoception improve the pragmatic search for biomarkers in psychiatry?. Front Psychiatry.

[3] Khalsa S, Rudrauf D, Feinstein J, Tranel D (2009). The pathways of interoceptive awareness. Nat. Neurosci..

[4] Craig A (2003). Interoception: the sense of the physiologial condition of the body. Curr. Opin. Neurobiol..

[5] Craig A (2009). How do you feel–now? The anterior insula and human awareness. Nature. Rev. Neurosci..

[6] Dakanalis A (2016). Body-image distortion in anorexia nervosa. Nat. Rev. Dis. Prim..

[7] Klabunde M, Acheson D, Boutelle KN, Matthews S, Kaye W (2013). Interoceptive sensitivity deficits in women recovered from bulimia nervosa. Eat. Behav..

[8] Badoud D, Tsakiris M (2017). From the body’s viscera to the body’s image: Is there a link between interoception and body image concerns?. Neurosci. Biobehav. Rev..

[9] Martin E, Dourish C, Rotshtein P, Spetter M, Higgs S (2019). Interoception and disordered eating: a systematic review. Neurosci. Biobehav. Rev..

[10] Craig AD (2002). How do you feel? Interoception: the sense of the physiological condition of the body. Nat. Rev. Neurosci..

[11] Ceunen E, Vlaeyen J, Van Diest I (2016). On the origin of interoception. Front Psychol..

[12] Brown T (2017). Psychometric evaluation and norms for the Multidimensional Assessment of Interoceptive Awareness (MAIA) in a clinical eating disorders sample. Eur. Eat. Disord. Rev..

[13] Jenkinson P, Taylor L, Laws K (2018). Self-reported interoceptive deficits in eating disorders: a meta-analysis of studies using the eating disorder inventory. J. Psychosom. Res..

[14] Papezova H, Yamamotova A, Uher R (2005). Elevated pain threshold in eating disorders: physiological and psychological factors. J. Psychiatr. Res..

[15] Lautenbacher S, Pauls A, Strian F, Pirke K-M, Kreig J-C (1991). Pain sensitivity in anorexia nervosa and bulimia nervosa. Biol. Psychiatry.

[16] de Zwaan M, Biener D, Bach M, Wiesnagrotzki S, Stacher G (1996). Pain sensitivity, alexithymia, and depression in patients with eating disorders: are they related?. J. Psychosom. Res..

[17] Stein D (2003). Pain perception in recoverd bulimia nervosa patients. Int J. Eat. Disord..

[18] Geliebter A (1988). Gastric distension and gastric capacity in relation to food intake in humans. Physiol. Behav..

[19] Walsh B, Zimmerli E, Devlin M, Guss J, Kissileff H (2003). A disturbance of gastric function in bulimia nervosa. Biol. Pschiatry..

[20] Pollatos O, Georgiou E (2016). Normal interoceptive accuracy in women with bulimia nervosa. Psychiatr. Res..

[21] Bohon C, Stice E (2012). Negative affect and neural response to palatable food intake in bulimia nervosa. Appetite.

[22] Monteleone A (2017). Altered processing of rewarding and aversive basic taste stimuli in symptomatic women with anorexia nervosa and bulimia nervosa: an fMRI study. J. Psychiatr. Res..

[23] Simon J (2016). Neural signature of food reward processing in bulimic-type eating disorders. Soc. Cogn. Affect Neurosci..

[24] Balodis I, Potenza M (2015). Anticipatory reward processing in addicted populations: a focus on the monetary incentive delay task. Biol. Psychiatry.

[25] Knutson B, Fong GW, Adams CM, Varner JL, Hommer D (2001). Dissociation of reward anticipation and outcome with event-related fMRI. Neuroreport.

[26] Garfinkel S (2016). Discrepancies between dimensions of interoception in autism: Implications for emotion and anxiety. Biol. Pschiatry..

[27] Lovero KL, Simmons A, Aron J, Paulus M (2009). Anerior insular cortex anticipates impending stimulus significance. Neuroimage.

[28] Lucas M, Anderson L, Bolling D, Pelphrey K, Kaiser M (2015). Dissociating the neural correlates of experiencing and imagining affective touch. Cereb. Cortex..

[29] Haber S, Knutson B (2010). The reward circuit: Linking primate anatomy and human imaging. Neuropsychopharm.

[30] Phillips M, Drevets WC, Rauch SL, Lane R (2003). Neurobiology of emotion perception I: The neural basis of normal emotion perception. Biol. Psych..

[31] Fudge J, Breibart M, Danish M, Pannoni V (2005). Insular and gustatory inputs to the caudal ventral striatum in primates. J. Comp. Neurol..

[32] Graybiel A (1997). The basal ganglia and cognitive pattern generators. Schizophr. Bull..

[33] Robbins T, Everitt B (1996). Neurobehavioural mechanisms of reward and motivation. Curr. Opin. Neurobiol..

[34] Berridge K, Ho C, Richard J, DiFeliceantonio A (2010). The tempted brain eats: Pleasure and desire circuits in obesity and eating disorders. Brain Res..

[35] Volkow N (2006). Cocaine cues and dopamine in dorsal striatum: mechanism of craving in cocaine addiction. J. Neurosci..

[36] Aron A (2005). Reward, motivation, and emotion systems associated with early-stage intense romantic love. J. Neurophysiol..

[37] Volkow ND (2002). “Nonhedonic” food motivation in humans involves dopamine in the dorsal striatum and methylephenidate amplifies this effect. Synapse.

[38] Wierenga C (2014). Are extremes of consumption in eating disorders related to an altered balance between reward and inhibition?. Front Behav. Neurosci..

[39] Berner L, Marsh R (2014). Frontostriatal circuits and the development of bulimia nervosa. Front Behav. Neurosci..

[40] Brooks S, Rask-Andersen M, Benedict C, Schioth H (2012). A debate on current eating disorder diagnoses in light of neurobiological findings: is it time for a spectrum model?. BMC Psychiatry.

[41] Wu M (2016). Reward-related decision making in eating and weight disorders: a systematic review and meta-analysis of the evidence from neuropsychological studies. Neurosci. Biobehav Rev..

[42] Oberndorfer T (2013). Altered insula response to sweet taste processing after recovery from anorexia and bulimia nervosa. Am. J. Psychiatry.

[43] Ely A (2017). Response in taste circuitry is not modulated by hunger and satiety in women remitted from bulimia nervosa. J. Abnorm Psychol..

[44] Frank G (2006). Altered brain activity in women recovered from bulimic type eating disorders after a glucose challenge. A pilot study. Int J. Eat. Disord..

[45] Wagner A (2015). Altered sensitization patterns to sweet food stimuli in patients recovered from anorexia and bulimia nervosa. Psychiatr. Res..

[46] Bohon C, Stice E (2011). Reward abnormalities among women with full and subthreshold bulimia nervosa: a functional magnetic resonance imaging study. Int J. Eat. Disord..

[47] Brooks S (2011). Differential neural responses to food images in women with bulimia versus anorexia nervosa. PLoS ONE.

[48] Berner L (2019). Altered anticipation and processing of aversive interoceptive experience among women remitted from bulimia nervosa. Neuropsychopharm.

[49] Frank G, Reynolds J, Shott M, O’Reilly R (2011). Altered temporal difference learning in bulimia nervosa. Biol. Psych..

[50] Berner L (2018). Altered interoceptive activation before, during, and after aversive breathing load in women remitted from anorexia nervosa. Psychol. Med..

[51] Bischoff-Grethe A (2018). Neural hypersensitivity to pleasant touch in women remitted from anorexia nervosa. Transl. Psychiatry.

[52] van Stralen H (2014). Affective touch modulates the rubber hand illusion. Cognition.

[53] Crucianelli L, Metcalf N, Fotopoulou A, Jenkinson P (2013). Bodily pleasure matters: velocity of touch modulates body ownership during the rubber hand illusion. Front Psychol..

[54] D’Alessandro G, Cerritelli F, Cortelli P (2016). Sensitization and interoception as key neurological concepts in osteopathy and other manual medicines. Front Neurosci..

[55] McGlone F, Wessberg J, Olausson H (2014). Discriminative and affective touch: sensing and feeling. Neuron.

[56] Suzuki K, Garfinkel SN, Critchley HD, Seth A (2013). Multisensory integration across exteroceptive and interoceptive domains modulates self-experience in the rubber-hand illusion. Neuropsychologia.

[57] Tsakiris M (2017). The multisensory basis of the self: From body to identity to others [Formula: see text]. Q J. Exp. Psychol..

[58] Marshall A, Sharma M, Marley K, Olausson H, McGlone F (2019). Spinal signalling of C-fiber mediated pleasant touch in humans. Elife.

[59] Cassin S, von Ranson K (2005). Personality and eating disorders: a decade in review. Clin. Psycho. Rev..

[60] Fassino S, Amianto F, Gramaglia C, Facchini F, Abbate Daga G (2004). Temperament and character in eating disorders: ten years of studies. Eat. Weight Disord..

[61] Klump K (2004). Personality characteristics of women before and after recovery from an eating disorder. Psych. Med..

[62] Wagner A (2006). Personality traits after recovery from eating disorders: Do subtypes differ?. Int. J. Eat. Disord..

[63] Sheehan DV (1998). The Mini-International Neuropspychiatric Interview (M.I.N.I.): the development and validation of a structured diagnostic psychiatric interview for DSM-IV and ICD-10. J. Clin. Psychiatry.

[64] Garner, D. M. *Eating Disorder Inventory-2 Professional Manual* (Psychological Assessment Resources, Inc., Odessa, FL, 1991).

[65] Spielberger, C., Gorsuch, R. & Lushene, R. *STAI Manual for the State Trait Anxiety Inventory* (Consulting Psychologists Press, Palo Alto, CA, 1970).

[66] Cloninger, C. R., Przybeck, T. R., Svrakic, D. M. & Wetzel, R. D. *The Temperament and Character Inventory (TCI): A Guide to its Development and Use*, 19–28 (Center for Psychobiology of Personality, Washington University, St. Louis, MO, 1994).

[67] Beck AT, Ward M, Mendelson M, Mock J, Erbaugh J (1961). An Inventory for measuring depression. Arch. Gen. Psychiatry.

[68] Löken L, Wessberg J, Morrison I, McGlone F, Olausson H (2009). Coding of pleasant touch by unmyelinated afferents in humans. Nat. Neurosci..

[69] Olausson H (2002). Unmyelinated tactile afferents signal touch and project to insular cortex. Nat. Neurosci..

[70] Vallbo A, Olausson H, Wessberg J, Norrsell U (1993). A system of unmyelinated afferents for innocuous mechanoreception in the human skin. Brain Res..

[71] May A, Stewart J, Migliorini R, Tapert S, Paulus MP (2013). Methamphetamine dependent individuals show attenuated brain response to pleasant interoceptive stimuli. Drug Alcohol Depend..

[72] Migliorini R, Stewart J, May A, Tapert S, Paulus MP (2013). What do you feel? Adolescent drug and alcohol users show altered brain response to pleasant interoceptive stimuli. Drug Alcohol Depend..

[73] Stewart J, May AC, Tapert S, Paulus M (2015). Hyperactivation to pleasant interoceptive stimuli characterizes the transition to stimulant addiction. Drug Alcohol Depend..

[74] Desikan R (2006). An automated labeling system for subdividing the human cerebral cortex on MRI scans into gyral based regions of interest. Neuroimage.

[75] Eklund A, Nichols T, Knutsson H (2016). Cluster failure: Why fMRI inferences for spatial extent have inflated false-positive rates. Proc. Natl Acad. Sci. USA.

[76] Hothorn T, Bretz F, Westfall P (2008). Simultaneous inference in general parametric models. Biom. J..

[77] Huber P (1964). Robust estimation of location parameter. Ann. Math. Stats..

[78] Nichols T, Brett M, Anderson J, Wager T, Poline J (2005). Valid conjunction inference with the minimum statistic. Neuroimage.

[79] Riva G, Dakanalis A (2018). Altered processing and integration of multisensory bodily representations and signals in eating disorders: a possible path toward the understanding of their underlying causes. Front Hum. Neurosci..

[80] Riva G (2018). The neuroscience of body memory: from the self through the space to the others. Cortex.

[81] Hohwy, J. *The Predictive Mind* (Oxford University Press, Oxford, 2013).

[82] Herbert, B. & Pollatos, O. in *The Interoceptive Basis of the Mind* (eds Tsakiris, M. & De Preester, H.) (Oxford University Press, NewYork, 2018).

[83] Alexi J, Palermo R, Rieger E, Bell J (2019). Evidence for a perceptual mechanism relating body size misperception and eating disorder symptoms. Eat. Weight Disord..

[84] Mölbert S (2017). Depictive and metric body size estimation in anorexia nervosa and bulimia nervosa: a systematic review and meta-analysis. Clin. Psychol. Rev..

[85] Brovelli A, Nazarian B, Meunier M, Boussaoud D (2011). Differential roles of caudate nucleus and putamen during instrumental. Neuroimage.

[86] Mueller S, Wang D, Pan R, Holt D, Liu H (2015). Abnormalities in hemispheric specialization of caudate nucleus connectivity in schizophrenia. JAMA Psychiatry.

[87] Marsh R (2009). Deficient activity in the neural systems that mediate self-regulatory control in bulimia nervosa. Arch. Gen. Psychiatry.

[88] Skunde M (2016). Neural signature of behavioural inhibition in women with bulimia nervosa. J. Psychiatr. Neurosci..

[89] Wagner A (2010). Altered striatal response to reward in bulimia nervosa after recovery. Int. J. Eat. Disord..

[90] Coutinho J (2015). Volumetric alterations in the nucleus accumbens and caudate nucleus in bulimia nervosa: a structural magnetic resonance imaging study. Int. J. Eat. Disord..

[91] Amianto F (2013). Brain volumetric abnormalities in patients with anorexia and bulimia nervosa: a voxel-based morphometry study. Psychiatr. Res..

[92] Berner, L. et al. Subcortical shape abnormalities in bulimia nervosa. *Biol. Psychiatry Cogn. Neurosci. Neuroimaging* **4**, 1070–1079 (2019). 10.1016/j.bpsc.2018.12.011 PMC6609503 30846367

[93] Davidovic M, Starck G, Olausson H (2019). Processing of affective and emotionally neutral tactile stimuli in the insular cortex. Dev. Cogn. Neurosci..

[94] Knutson B, Heinz A (2015). Probing psychiatric symptoms with the monetary incentive delay task. Biol. Psychiatry.

[95] Paulus M, Feinstein J, Khalsa S (2019). An active inference approach to interoceptive psychopathology. Ann. Rev. Clin. Psychol..

[96] Pearson C, Chester D, Powell D, Wonderlich S, Smith G (2016). Investigating the reinforcing value of binge anticipation. Int. J. Eat. Disord..

[97] Smith K, Mason T, Peterson C, Pearson C (2018). Relationships between eating disorder-specific and transdiagnostic risk factors for binge eating: an integrative moderated mediation model of emotion regulation, anticipatory reward, and expectancy. Eat. Behav..

[98] Berner LA (2017). Temporal associations between affective instability and dysregulated eating behavior in bulimia nervosa. J. Psychiatr. Res..

[99] Wonderlich J (2017). The relation between craving and binge eating: Integrating neuroimaging and ecological momentary assessment. Appetite.

[100] Bailer U (2013). Interaction between serotonin transporter and dopamine D2/D3 receptor radioligand measures is associated with harm avoidant symptoms in anorexia and bulimia nervosa. Psych. Res. Neuroimaging..

[101] Frank G (2005). Increased dopamine D2/D3 receptor binding after recovery from anorexia nervosa measured by positron emission tomography and [11C]raclopride. Biol. Psychiatry.

[102] Li S (2017). Novelty seeking and reward dependence-related large-scale brain networks functional connectivity variation during salience expectancy. Hum. Brain Mapp..

[103] Liljencrantz J, Olausson H (2014). Tactile C fibers and their contributions to pleasant sensations and to tactile allodynia. Front Behav. Neurosci..

[104] McGlone F (2012). Touching and feeling: differences in pleasant touch processing between glabrous and hairy skin in humans. Eur. J. Neurosci..

[105] Robinson J (2012). The functional connectivity of the human caudate: an application of meta-analytic connectivity modeling with behavioral filtering. Neuroimage.

[106] Ghaziri J (2018). Subcortical structural connectivity of insular subregions. Sci. Rep..

[107] Morrison I (2016). ALE meta-analysis reveals dissociable networks for affective and discriminative aspects of touch. Hum. Brain Mapp..

[108] Stewart J, Huavinett A, May A, Davenport P, Paulus M (2015). Do you feel alright? Attenuated neural processing of aversive interoceptive stimuli in current stimulant users. Psychophysiology.

