# Supplementary material for: Increased anticipatory brain response to pleasant touch in women remitted from bulimia nervosa
Source: Transl Psychiatry. 2020 Jul 16;10:236. doi: 10.1038/s41398-020-00916-0 (PMC7363900; doi:10.1038/s41398-020-00916-0)
Supplement: Supplementary file 1 — Supplementary Information [file 41398_2020_916_MOESM1_ESM.docx]

**Supplemental Information**

**Materials and Methods**

*Image acquisition.* Neuroimaging data were acquired using T2* weighted echo planar imaging (EPI) on a 3T General Electric Discovery MR 750 (Milwaukee, WI) (252 volumes, TR=2 s, TE=30 ms, flip angle=90°, FOV 24 cm, 64×64 matrix, 3.75×3.75 mm in-plane resolution, 40 3.0 mm ascending interleaved axial slices). High-resolution T1-weighted FSPGR anatomical images (flip angle=8°, 256×256 matrix, 172 1 mm sagittal slices, TR=8.1 s, TE=3.17 ms, 1×1mm in-plane resolution) were acquired to permit activation localization and spatial normalization. EPI-based field maps corrected susceptibility-induced geometric distortions.

### *Image preprocessing.* Functional images were preprocessed and analyzed using Analysis of Functional NeuroImages (AFNI)^1^ and FSL^2^ software. EPIs were slice-time corrected, motion-corrected, and aligned to high-resolution anatomical images using AFNI’s align_epi_anat.py.^3^ Time points with isolated head movements not corrected by coregistration were censored. T1-weighted images were skull-stripped with FreeSurfer’s mri_watershed^4^ and registered to the MNI-152 atlas using affine transform followed by nonlinear refinement using FSL’s FLIRT and FNIRT^5, 6^. Functional data were aligned to standard space, resampled to 3 mm isotropic voxels, and smoothed with a 4.2 mm FWHM Gaussian kernel. For each participant, AFNI’s 3dDeconvolve was used to determine activation related to the soft touch paradigm. Four task regressors (anticipation palm, anticipation forearm, soft touch palm, soft touch forearm) were convolved with a modified hemodynamic response function. Six motion regressors and 5 noise regressors of orders of polynomials trends (baseline, linear, quadratic, etc.) were included as covariates of no interest. Following deconvolution, the four task beta regressors were converted to percent signal change.

Supplemental Table 1. Participant demographics and characteristics.

| **Characteristic** | **CW (N=25)**  **Mean (SD)** | **RBN (N=23)**  **Mean (SD)** | **df** | **t or χ^2^** | **p-value** | **Cohen’s *d*** | **95% CI** | |
| --- | --- | --- | --- | --- | --- | --- | --- | --- |
|  |  |  |  |  |  |  | **Lower bound** | **Upper bound** |
| Age (years) | 25.6 (7.3) | 27.2 (4.0) | 37.4 | -0.9 | 0.4 | 0.27 | -4.9 | 1.9 |
| Education (years)^b^ | 15.2 (1.4) | 16.5 (1.4) | 44.1 | -3,1 | 0.004 | 0.93 | -2.1 | -0.4 |
| Current BMI | 22.2 (1.8) | 22.0 (2.0) | 45.0 | 0.3 | 0.8 | 0.11 | -0.9 | 1.3 |
| Lowest BMI^a^ | 19.9 (1.1) | 17.6 (2.6) | 28.9 | 4.0 | <0.001 | 1.2 |  | 3.5 |
| BN Duration (months) | N/A | 33.3 (22.8) |  |  |  |  |  |  |
| BN Remission (months)^b^ | N/A | 82.0 (51.8) |  |  |  |  |  |  |
| Worst Past Binge Eating Freq (episodes/week) | N/A | 21.6 (18.1) |  |  |  |  |  |  |
| Worst Past Vomiting Freq (episodes/week) | N/A | 24.9 (22.3) |  |  |  |  |  |  |
| EDI Drive for Thinness | 0.2 (1.0) | 0.6 (1.2) | 42.4 | -1.3 | 0.2 | 0.36 | -1.1 | 0.2 |
| EDI Bulimia | 0.1 (0.4) | 0.3 (0.6) | 36.5 | -1.5 | 0.2 | 0.39 | -0.5 | 0.1 |
| EDI Body Dissatisfaction | 0.7 (1.3) | 2.2 (3.0) | 30.0 | -2.3 | 0.03 | 0.65 | -2.9 | -0.2 |
| EDI Ineffectiveness | 0.6 (1.2) | 0.7 (1.3) | 45.4 | -0.1 | 0.9 | 0.08 | -0.8 | 0.7 |
| EDI Perfectionism | 5.0 (3.4) | 6.3 (3.4) | 45.8 | -1.4 | 0.2 | 0.38 | -3.3 | 0.6 |
| EDI Interpersonal Distrust | 0.4 (1.0) | 0.8 (1.8) | 32.8 | -0.9 | 0.4 | 0.27 | -1.2 | 0.5 |
| EDI Interoceptive Awareness | 0.3 (0.8) | 0.2 (0.5) | 39.2 | 0.5 | 0.6 | 0.15 | -0.3 | 0.5 |
| BDI-II | 0.3 (0.7) | 2.2 (2.6) | 24.7 | -3.3 | 0.002 | 1.0 | -3.1 | -0.7 |
| State Anxiety (STAI-S) | 22.6 (3.3) | 30.3 (6.7) | 31.6 | -5.0 | <0.001 | 1.5 | -10.8 | -4.6 |
| Trait Anxiety (STAI-T) | 23.2 (2.8) | 30.4 (7.9) | 27.1 | -4.2 | <0.001 | 1.2 | -10.8 | -3.7 |
| TCI Harm Avoidance^a^ | 7.3 (4.4) | 14.0 (8.1) | 33.7 | -3.5 | 0.001 | 1.0 | -10.6 | -2.9 |
| TCI Novelty Seeking^a^ | 20.1 (4.7) | 19.3 (5.9) | 41.9 | 0.5 | 0.6 | 0.15 | -2.3 | 4.0 |
| TCI Reward Dependence^a^ | 16.8 (3.4) | 17.7 (2.9) | 44.6 | -1.0 | 0.3 | 0.28 | -2.8 | 1.0 |
| Lifetime Diagnoses (No.) |  |  |  |  |  |  |  |  |
| Anorexia Nervosa | 0 | 11 | 1 | 15.0 | <0.001 |  |  |  |
| Major Depressive Disorder^b^ | 0 | 15 | 1 | 23.7 | <0.001 |  |  |  |
| Any Anxiety Disorder^b, c^ | 0 | 2 | 1 | 1.3 | 0.3 |  |  |  |
| Obsessive Compulsive Disorder^b^ | 0 | 1 | 1 | 1.1 | 0.5 |  |  |  |
| Any Substance Use Disorder^b, d^ | 0 | 7 | 1 | 8.9 | 0.003 |  |  |  |

Note: Entries are of the form mean (SD: standard deviation). Statistical comparisons were by means of Welch’s t-tests, and Cohen’s *d* effect sizes are reported. BDI-II: Beck Depression Inventory II; BMI: body mass index; CW: healthy comparison women; N/A: not applicable; EDI: Eating Disorders Inventory; RBN: women remitted from bulimia nervosa; STAI: Spielberger State-Trait Anxiety Inventory; TCI: Temperament and Character Inventory. ^a^One CW did not complete this assessment. ^b^One RBN did not complete this assessment. ^c^Defined as having had at least one prior episode of panic disorder, posttraumatic stress disorder, generalized anxiety disorder, or any anxiety disorder not otherwise specified. ^d^Defined as any history of abuse or dependence per DSM-IV criteria.

Supplemental Table 2. VAS ratings on the experience of soft touch of the forearm or palm.

|  | **CW (n=25)**  **Mean (SD)** | **RBN (n=22)**  **Mean (SD)** | **df** | **t** | **p-value** | **Cohen’s *d*** | **95% CI** | |
| --- | --- | --- | --- | --- | --- | --- | --- | --- |
|  |  |  |  |  |  |  | **Lower bound** | **Upper bound** |
| Pre-Scan Forearm |  |  |  |  |  |  |  |  |
| Pleasant | 46.5 (26.7) | 50.1 (27.0) | 44.1 | -0.5 | 0.6 | 0.13 | -19.4 | 12.2 |
| Unpleasant | 6.9 (15.2) | 14.2 (21.8) | 36.9 | -1.3 | 0.2 | 0.39 | -18.6 | 3.9 |
| Intense | 6.3 (14.6) | 13.0 (19.4) | 38.7 | -1.3 | 0.2 | 0.39 | -16.9 | 3.5 |
| Post-Scan Forearm^a^ |  |  |  |  |  |  |  |  |
| Pleasant | 46.2 (27.2) | 52.4 (33.4) | 40.6 | -0.7 | 0.5 | 0.20 | -24.4 | 12.0 |
| Unpleasant | 4.5 (10.6) | 6.6 (10.7) | 43.6 | -0.7 | 0.5 | 0.20 | -8.5 | 4.2 |
| Intense | 3.8 (9.9) | 10.8 (21.4) | 29.0 | -1.4 | 0.2 | 0.42 | -17.2 | 3.2 |
| Pre-Post Scan Forearm^a^ |  |  |  |  |  |  |  |  |
| Pleasant | 2.1 (19.7) | -2.2 (21.2) | 42.9 | 0.7 | 0.5 | 0.20 | -7.8 | 16.5 |
| Unpleasant | 1.7 (16.5) | 7.6 (23.9) | 36.9 | 1.0 | 0.3 | 0.29 | -18.3 | 6.5 |
| Intense | 2.8 (13.0) | 2.3 (15.7) | 41.0 | 0.1 | 0.9 | 0.03 | -8.2 | 9.1 |
| Pre-Scan Palm |  |  |  |  |  |  |  |  |
| Pleasant | 49.1 (21.7) | 59.1 (22.5) | 43.8 | -1.5 | 0.1 | 0.45 | -23.0 | 3.1 |
| Unpleasant | 6.6 (14.7) | 6.5 (11.6) | 44.5 | 0.04 | 1.0 | 0.01 | -7.6 | 7.9 |
| Intense | 7.4 (15.7) | 9.1 (18.4) | 41.6 | -0.3 | 0.7 | 0.10 | -11.8 | 8.4 |
| Post-Scan Palm^a^ |  |  |  |  |  |  |  |  |
| Pleasant | 48.9 (24.8) | 57.9 (28.9) | 41.7 | -1.1 | 0.3 | 0.33 | -25.1 | 7.1 |
| Unpleasant | 2.7 (6.0) | 3.9 (9.2) | 35.5 | -0.5 | 0.6 | 0.15 | -5.9 | 3.5 |
| Intense | 2.8 (7.7) | 14.1 (25.3) | 24.5 | -2.0 | 0.1 | 0.62 | -22.9 | 0.3 |
| Pre-Post Scan Palm^a^ |  |  |  |  |  |  |  |  |
| Pleasant | 0.2 (20.6) | 1.2 (24.0) | 41.5 | -0.2 | 0.9 | 0.04 | 0.2 | 1.2 |
| Unpleasant | 3.7 (16.3) | 2.6 (14.3) | 43.9 | 0.2 | 0.8 | 0.07 | -8.0 | 10.2 |
| Intense | 5.0 (16.0) | -5 (19.3) | 40.9 | 1.9 | 0.07 | 0.56 | -0.7 | 20.5 |

Note: Entries are of the form mean (standard deviation). Statistical comparisons were by means of Welch’s t-tests, and Cohen’s *d* effect sizes are reported. CW: healthy comparison women RBN: women remitted from bulimia nervosa; ^a^One CW failed to complete the post-scan VAS ratings.

| Supplemental Table 3. Voxelwise linear mixed effects analysis results for the soft touch paradigm showing main effects of Condition (Anticipation, Soft Touch) and Location (Palm, Forearm), and interactions of Group (CW, RAN) x Condition. | | | | | | | | | | | | | |
| --- | --- | --- | --- | --- | --- | --- | --- | --- | --- | --- | --- | --- | --- |
|  | | | | | | | | **Post Hoc Comparisons** | | | | | |
| **Region** | **L/R** | **Volume (voxels)** | **x** | **y** | | **z** | **F value** | **Comparison** | | **z** | | **p(FDR)** | |
| **GROUP** | | | | | | | | | | | | | |
| Precuneus | L | 73 | -21 | -57 | | 51 | 12.23 | RBN > CW | | 3.837 | | <0.001 | |
| Inferior Frontal Gyrus | L | 56 | -47 | 12 | | 23 | 9.03 | RBN > CW | | 4.058 | | <0.001 | |
| Inferior Parietal Lobule | R | 52 | 37 | -36 | | 40 | 9.62 | RBN > CW | | 3.486 | | <0.001 | |
| Inferior Frontal Gyrus | L | 46 | -45 | 1 | | 38 | 8.2 | RBN > CW | | 3.205 | | 0.001 | |
| **CONDITION*** | | | | | | | | | | | | | |
| Postcentral Gyrus | L | 3454 | -28 | -18 | | 28 | 52.68 | Soft Touch > Anticipation | | 7.848 | | <0.001 | |
| Insula | R | 2890 | 41 | -21 | | 16 | 58.38 | Soft Touch > Anticipation | | 8.889 | | <0.001 | |
| Cuneus | R | 414 | 10 | -76 | | 12 | 31.66 | Soft Touch > Anticipation | | 6.045 | | <0.001 | |
| Precuneus | R | 265 | 1 | -54 | | 32 | 31.86 | Soft Touch > Anticipation | | 5.205 | | <0.001 | |
| Superior Frontal Gyrus | R | 182 | 18 | 32 | | 46 | 23.59 | Soft Touch > Anticipation | | 5.984 | | <0.001 | |
| Inferior Frontal Gyrus | R | 159 | 40 | 38 | | 1 | 29.67 | Soft Touch > Anticipation | | 6.735 | | <0.001 | |
| Superior Frontal Gyrus | L | 85 | -17 | 39 | | 42 | 23.81 | Soft Touch > Anticipation | | 5.177 | | <0.001 | |
| Middle Frontal Gyrus | L | 74 | -23 | 21 | | 49 | 19.25 | Soft Touch > Anticipation | | 4.785 | | <0.001 | |
| Parahippocampal Gyrus | L | 66 | -28 | -28 | | -20 | 21.77 | Soft Touch > Anticipation | | 6.052 | | <0.001 | |
| Middle Frontal Gyrus | R | 41 | 21 | -13 | | 66 | 24.09 | Soft Touch > Anticipation | | -5.559 | | <0.001 | |
| Superior Frontal Gyrus | L | 36 | -10 | 59 | | 24 | 23.67 | Soft Touch > Anticipation | | 4.718 | | <0.001 | |
| Middle Frontal Gyrus | L | 33 | -44 | 13 | | 44 | 25.73 | Soft Touch > Anticipation | | 5.209 | | <0.001 | |
| Middle Temporal Gyrus | L | 32 | -65 | -18 | | -12 | 24.41 | Soft Touch > Anticipation | | 5.774 | | <0.001 | |
| Precuneus | R | 28 | 24 | -62 | | 42 | 21.25 | Soft Touch > Anticipation | | 4.634 | | <0.001 | |
| Cingulate Gyrus | L | 26 | -6 | -9 | | 33 | 30.2 | Soft Touch > Anticipation | | 5.466 | | <0.001 | |
| Superior Temporal Gyrus | L | 24 | -62 | -14 | | 1 | 17.34 | Soft Touch > Anticipation | | 5.023 | | <0.001 | |
| Cuneus | L | 15 | -8 | -83 | | 10 | 15.51 | Soft Touch > Anticipation | | 3.827 | | <0.001 | |
| Postcentral Gyrus | L | 15 | -64 | -24 | | 20 | 18.04 | Soft Touch > Anticipation | | 4.714 | | <0.001 | |
| Middle Temporal Gyrus | L | 14 | -55 | 6 | | -22 | 19.87 | Soft Touch > Anticipation | | 4.930 | | <0.001 | |
| Medial Frontal Gyrus | L | 13 | -4 | 63 | | 0 | 15.32 | Soft Touch > Anticipation | | 4.597 | | <0.001 | |
| **LOCATION** | | | | | | | | | | | | | |
| Postcentral Gy | R | 377 | 42 | -22 | | 55 | 53.61 | Palm > Forearm | | 5.91 | | <0.001 | |
| **GROUP x CONDITION** | | | | | | | | | | | | | |
| Cuneus | R/L | 160 | 8 | -80 | 11 | | 9.75 | CW: Soft Touch > Anticipation | 7.07 | | <0.001 | |  |
|  |  |  |  |  |  | |  | RBN: Soft Touch > Anticipation | 3.30 | | 0.006 | |  |
| Precuneus | L | 97 | -24 | -70 | 34 | | 12.21 | CW: Soft Touch > Anticipation | 3.50 | | 0.003 | |  |
| Superior Frontal Gyrus | R | 67 | 23 | 14 | 48 | | 10.2 | CW: Soft Touch > Anticipation | 4.59 | | <0.001 | |  |
|  |  |  |  |  |  | |  | Anticipation: RBN > CW | 2.82 | | 0.029 | |  |
| Note: Center of mass coordinates reported in MNI space. Correction for multiple comparisons was determined with Monte-Carlo simulations (via AFNI’s 3dClustSim) to guard against false positives. Post hoc analyses were conducted using glht from the multcomp package in R to calculate general linear hypotheses using Tukey’s all-pair comparisons, and p-values were Bonferroni adjusted. CW: healthy comparison women; L: left; R: right; RBN: women remitted from bulimia nervosa. *Because a main effect of condition encompassed nearly the whole brain, a more stringent threshold of p<0.001 (minimum cluster size 13 voxels) was used to better describe effects therein. | | | | | | | | | | | | | |

| Supplemental Table 4. Within-group associations of VAS and Self-Report ratings with neural activation within our *a priori* search regions of interest (insula, striatum). | | | | | | | | | |  |
| --- | --- | --- | --- | --- | --- | --- | --- | --- | --- | --- |
| **Variable** | **Region** | **L/R** | **Volume**  **(voxels)** | **X** | **Y** | **Z** | **t** | **r** | **p** | **GxC Overlap (voxels)** |
| **Women Remitted from Bulimia Nervosa** | | | | | | | | | |  |
| **TCI Harm Avoidance** |  |  |  |  |  |  |  |  |  |  |
| Anticipation Palm | Caudate | R | 64 | 12 | 8 | 15 | 4.08 | 0.65 | <0.001 | 15 |
| **TCI Reward Dependence** | |  |  |  |  |  |  |  |  |  |
| Soft Touch Forearm | Insula | L | 62 | -31 | 18 | 0 | 5.34 | 0.78 | <0.001 | 0 |
| **Illness Duration** | | | | | | | | | |  |
| Soft Touch Palm | Putamen | L | 35 | -22 | 6 | -4 | -4.39 | -0.67 | <0.001 | 0 |
| **Control Women** | | | | | | | | | | |
| **Pre-Scan Palm Pleasant** | |  |  |  |  |  |  |  |  |  |
| Anticipation Palm | Nucleus Accumbens | L | 38 | -14 | 12 | -7 | 4.32 | 0.69 | <0.001 | 0 |
| Note: Coordinates are reported as the center of mass. CW: healthy comparison women; L: left; R: right; RBN women remitted from bulimia nervosa. Significant clusters identified by exploratory within-group Huber robust regression or Poisson regression (for frequency count measures) analyses associating brain activity with VAS ratings and clinical measures within a priori search regions of the interest: the insula and striatum (voxel p<0.01, Bonferroni corrected p<0.0003). | | | | | | | | | |  |

Supplemental Figure 1. The soft touch continuous performance task


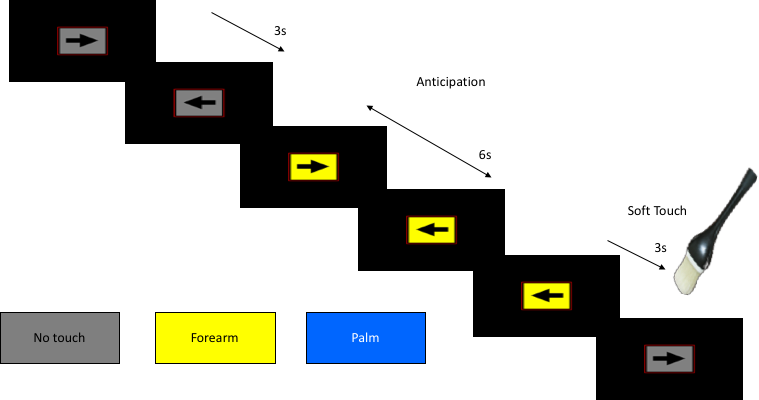


Supplemental Figure 2. Illustration of regions of interest used in the analysis including the striatum, that comprised the caudate, putamen, and nucleus accumbens (red), and the insula (orange).


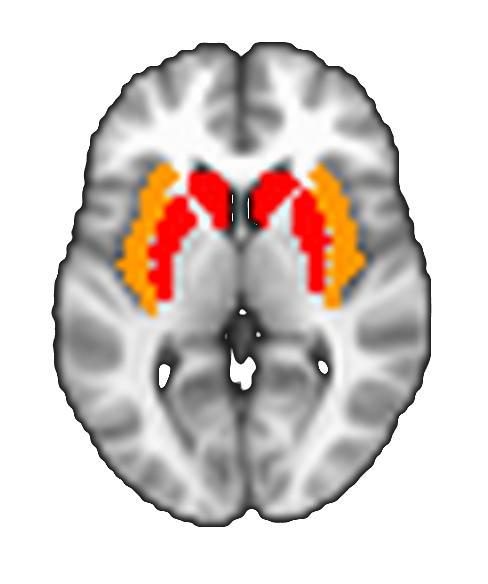

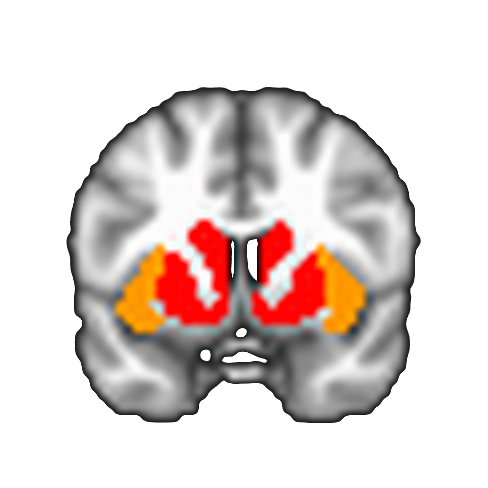


Y = 6

Z = 3

**References**

**1.** Cox R. AFNI: software for analysis and visualization of functional magnetic resonance neuroimages. *Comput Biomed Res*.**29**:162-173 (1996).

**2.** Jenkinson M, Beckmann C, Behrens T, Woolrich M, Smith S. FSL. *Neuroimage*.**62**(12):782-790 (2012).

**3.** Saad Z, et al. A new method for improving functional-to-structural MRI alignment using local Pearson correlation. *Neuroimage*.**44**(3):839-848 (2009).

**4.** Segonne F, et al. A hybrid approach to the skull stripping problem in MRI. *Neuroimage*.**22**(3):1060-1075 (2004).

**5.** Andersson J, Jenkinson M, Smith S. Non-linear registration, aka spatial normalisation. FMRIB technical report TR07JA2. <http://www.fmrib.ox.ac.uk/analysis/techrep/tr07ja2/tr07ja2.pdf>. (2010).

**6.** Jenkinson M, Smith S. A global optimisation method for robust affine registration of brain images. *Med Image Anal*.**5**(2):143-156 (2001).
